# All-arthroscopic AMIC^®^ (AT-AMIC^®^) technique with autologous bone graft for talar osteochondral defects: clinical and radiological results

**DOI:** 10.1007/s00167-016-4318-4

**Published:** 2016-09-12

**Authors:** Federico Giuseppe Usuelli, Riccardo D’Ambrosi, Camilla Maccario, Michele Boga, Laura de Girolamo

**Affiliations:** 1 IRCCS Istituto Ortopedico Galeazzi, Milan, Italy; 2 Università degli Studi di Milano, Milan, Italy; 3 Laboratorio di Biotecnologie Applicate all’Ortopedia, IRCCS Istituto Ortopedico Galeazzi, Milan, Italy

**Keywords:** Osteochondral talar lesions, Autologous matrix-induced chondrogenesis, Autologous bone graft, Ankle arthroscopy, MOCART, Cartilage healing

## Abstract

**Purpose:**

Autologous Matrix-Induced Chondrogenesis (AMIC^®^) is known to provide satisfactory clinical results for the treatment of knee, hip, and ankle cartilage lesions. The purpose of this study was to evaluate clinical and radiological outcomes of patients treated with a new all-arthroscopic AMIC^®^ (AT-AMIC^®^) technique with autologous bone graft for talar osteochondral defects at a follow-up of 24 months.

**Methods:**

Twenty patients underwent the AT-AMIC^®^ procedure and autologous bone graft for type III and IV talar osteochondral lesions. Patients were evaluated pre-operatively and at 6, 12, and 24 months post-operatively using the American Orthopedic Foot and Ankle Society (AOFAS) score, the visual analog scale, and the SF-12 (Short Form-12). Radiological assessment included computed tomography (CT), magnetic resonance imaging (MRI), and magnetic resonance observation of cartilage repair tissue (MOCART).

**Results:**

All scores significantly improved (*p* < 0.05) with respect to pre-operative values after 6 months. Further improvements were detected at 24 months (AOFAS, from 57.1 ± 14.9 before surgery to 86.6 ± 10.9 after 24 months; VAS, from 8.1 ± 1.4 to 2.5 ± 2.2; SF-12, from 29.9 ± 4.1 to 48.5 ± 6.9 and from 43.8 ± 2.9 to 53.1 ± 3.9, respectively, for Physical and Mental component score). Lesion area significantly reduced from 111.1 ± 43.2 mm^2^ pre-operatively to 76.9 ± 38.1 mm^2^ (*p* < 0.05) at final follow-up as assessed by CT, and from 154.1 ± 93.6 to 94.3 ± 61.3 mm^2^ (*p* < 0.05) as assessed by MRI. The mean MOCART score was 42.8 ± 23.5 points and 50.9 ± 24.9 points, respectively, at 12 and 24 months after surgery (*p* < 0.05).

**Conclusions:**

AT-AMIC^®^ with autologous bone grafting has proven to be a safe and effective minimal invasive technique, able to rapidly and significantly improve pain, function, and radiological healing of osteochondral talar lesions, with progressive further improvements up to 24 months. Orthopedic surgeons specialized in foot and ankle surgery should adopt the AT-AMIC^®^ technique for the treatment of osteochondral talar lesions, which proved to be effective and minimally invasive, avoiding malleolar osteotomy with a low risk of complications.

**Level of evidence:**

IV.

## Introduction

Among the different surgical techniques for the treatment of acute osteochondral and chronic severe lesions, Autologous Matrix-Induced Chondrogenesis (AMIC^®^) has proven to provide satisfactory results through medium-term follow-up in knee, hip, and talus [7, 13, 21, 25]. AMIC^®^ combines microfractures with the application of Chondro-Gide^®^, a porcine collagen type I/III bilayer matrix (Geistlich Surgery, Wolhusen, Switzerland), used to stabilize and protect the blood clot that results from microfracturing the subchondral bone. The AMIC^®^ technique effectively takes advantage of the ability of the resident bone marrow progenitor cells to regenerate articular cartilage. Usually AMIC^®^, for the treatment of osteochondral talar lesions (OCLTs), is performed in arthrotomy; however, in an attempt to minimize morbidity, we recently developed an all-arthroscopic AMIC^®^. This Arthroscopic-Talus Autologous Matrix-Induced Chondrogenesis (AT-AMIC^®^) technique has the advantages of smaller incisions, less soft tissue dissection, better visualization of the joint, and is predicted to provide quicker patient recovery, compared to the open technique [20].

The purpose of this study is thus to evaluate the clinical and radiological results of patients treated with the AT-AMIC^®^ and autologous bone graft for osteochondral defects of the ankle at a follow-up of 24 months. To our knowledge, this study is the first to report outcomes of this technique for the treatment of OCLTs.

## Materials and methods

Patients were recruited from among those presenting with OCLTs at our office between February 2012 and October 2013. OCLTs were diagnosed clinically and radiologically with magnetic resonance imaging (MRI) and computed tomography (CT). The inclusion criteria for the prospective case series of patients were: osteochondral lesion of the talus Type III and IV according to Berndt and Harty’s classification [2], age between 17 and 58 years, having reached skeletal maturity, ability to give informed consent, ability to understand the study protocol and to participate for its whole duration. Exclusion criteria were: previous surgical treatment of the affected ankle, arthritis of the ankle joint, kissing lesions, hemophilia, rheumatoid arthritis, diabetes, autoimmune disease, ongoing chemotherapy, radiation treatment or immunosuppression, pregnancy or lactation.

Of 27 patients screened for eligibility, 20 satisfied the inclusion and exclusion criteria and were enrolled in the study.

### Surgical technique

All surgical interventions were performed using the AT-AMIC^®^ technique [20]. Briefly, surgery was characterized by two different arthroscopic phases. First, after having achieved an adequate exposure through the use of a Hintermann™ spreader (Integra LifeSciences, Plainsboro, NJ) that allowed for sufficient joint distraction, the lesion was debrided with a shaver and prepared to receive the treatment. Microfractures are induced by a Chondro Pick (Arthrex, Naples, FL) on the healthy subchondral bone underneath the defect. This procedure is performed for the entire size of the lesion. Cancellous bone was harvested from the ipsilateral calcaneus with an accessory lateral approach on the calcaneus wall. The cancellous bone was introduced using the same cannula and impacted into the bony defect until having filled it completely. The second surgical step was performed in a dry condition, during which Chondro-Gide^®^ (Geistlich Surgery, Wolhusen, Switzerland), a porcine collagen type I/III matrix, was placed and fixed by synthetic fibrin glue (Tisseel^®^, Baxter, Baxter, USA) along the lesion edges. The Hintermann™ spreader was then removed and matrix stability within a normal ankle range of motion was verified.

Post-operative management included movement restriction of the ankle joint for 15 days to avoid matrix mobilization. To avoid algodystrophy syndrome, passive and active movement were then progressively allowed with no weight-bearing for up to 30 days [18].

### Clinical and radiological assessment

Each patient was evaluated pre-operatively, as well as at 6, 12, and 24 months. The evaluation included clinical and demographic parameters. Clinical evaluation consisted of subjective global pain assessment by the VAS pain score, while the intensity of pain, walking capacity, and activities of daily life were assessed by the AOFAS and SF-12 [9, 26, 27].

MRI and CT scan evaluations were also performed pre-operatively and at 6, 12, and 24 months post-operatively. The area of the lesions was defined and measured for each patient on the MRI and CT scan according to Choi [3] using coronal length (horizontal extension measured from the coronal image), sagittal length (horizontal extension measured from the sagittal image), depth (vertical extension measured from the sagittal image), and area (calculated with the ellipse formula as coronal length x sagittal length x 0.79). The magnetic resonance observation of cartilage repair Tissue (MOCART) [22] score was calculated on MRI, performed at 12 and 24 months after surgery. All radiological measurements were made using the standard tools of the institution’s Picture Archiving and Communication System (PACS). Clinical and radiographic evaluations were evaluated by two orthopedic surgeons, who were not directly involved in the surgical procedures. For the localization of the lesions, the 9-zone grid was used [4].

Detailed information about the surgical interventions was provided to all patients. All patients signed an informed consent form that detailed the surgical technique that would be performed. The patients were also informed about the rehabilitation protocol. The Institutional Review Board of IRCCS Ospedale San Raffaele of Milan approved this study (IRB Study Protocol: 49/INT/2016).

### Statistical analysis

The statistical analysis was performed by Matlab statistical toolbox version 2008 (MathWorks, Natick, MA, USA) for Windows at 32 bit, on a sample of 20 patients. Student *t* test was used to compare pre-operative and post-operative data. All the results were expressed as mean ± standard deviation. A *p* value <0.05 was considered statistically significant.

The estimated sample size for this study was obtained using the Bernoulli model, described as follows:

$$n_{c} = \frac{{z_{{\frac{\alpha }{2}}} \pi \left( {1 - \pi } \right)}}{{\varepsilon^{2} }}$$

with a z-score at 95 %, an error *ε* = 5 % and a prevalence of about 1 % [17] for this pathology: *π* = 1 %. Under these Hypotheses, the estimated sample size was equal to 15 patients.

In addition, our sample was increased to 20 patients, to preserve the statistic significance in the case of unexpected events.

## Results

All patients enrolled in the study and treated with AT-AMIC^®^ followed the study protocol. All lesions were filled with bone harvested from ipsilateral calcaneus. No associated procedures were performed at the time of index surgery. No infections occurred in the post-operative period and patients were clinically monitored during the follow-up at regular intervals. Only a single adverse effect occurred among all patients. In one case, 8 months after the first surgery, an anterior osteophyte due to hypertrophic proliferation that caused impingement and reduction in range of motion of the ankle, necessitated repeat arthroscopy which resulted in its removal and debridement. Two years after the patient was symptom-free, he had returned to sport activities and, as documented by CT and MRI, the lesion size appeared to be reduced.

Patient data are reported in Table 1. 55.0 % of patients were male and 45.0 % female, with a mean age of 36.1 ± 13.1 years and a mean lesion size of 111.1 ± 43.2 and 154.1 ± 93.6 mm^2^, as calculated from CT scans and MRI data, respectively. The mean pre-operative VAS score revealed severe baseline pain among included patients (8.1 ± 1.4) (Table 1).

**Table 1:** Population and lesion specific data

Age (years)	36.1 ± 13.1
Gender	11 M; 9 F
BMI (Kg/m^2^)	24.6 ± 2.7
Lesion localization	9 CM; 7 PM; 2 AM; 1 CL; 1 CC;

*CM* Centromedial*, PM* Posteromedial*, AM* Anteromedial, *CL* Centrolateral, *CC* Centrocentral

A significant improvement was observed in the VAS score which changed from the pre-operative score of 8.1 ± 1.4 to 4.8 ± 2.7 already after 6 months and reached 2.5 ± 2.2 at 24 months (*p* < 0.05, Table 2). The differences between pre-operative and 6-month AOFAS and SF-12 scores were also statistically significant. The AOFAS was 57.1 ± 14.9 initially and 71.5 ± 15.7 at 6 months (*p* < 0.05, Table 2). The SF-12 outcomes changed from baseline values of 29.9 ± 4.1 to 38.6 ± 8.1, and from 43.8 ± 2.9 to 50.9 ± 4.3, for Physical Component Score (PCS) and Mental Component Score (MCS), respectively, after 6 months. These functional outcomes continued improvement at the 24-month follow-up where the AOFAS score was 86.6 ± 10.9, PCS-SF-12 was 48.5 ± 6.9, and MCS-SF-12 53.1 ± 3.9. Improvements measured with the VAS, AOFAS, and SF-12 PCS were statistically significant both after 12 and 24 months with respect to the 6 month evaluations (*p* < 0.05, Table 2).

**Table 2:** Functional scores over 24 months

	Pre-op	6 months	12 months	24 months
VAS score	8.1 ± 1.4	4.8 ± 2.7^a^	2.9 ± 2.5^a,b^	2.5 ± 2.2^a,b^
AOFAS score	57.1 ± 14.9	71.5 ± 15.7^a^	80.0 ± 14.5^a,b^	86.6 ± 10.9^a,b,c^
SF 12 PCS score	29.9 ± 4.1	38.6 ± 8.1^a^	43.5 ± 9.8^a,b^	48.5 ± 6.9^a,b^
SF 12 MCS score	43.8 ± 2.9	50.9 ± 4.3^a^	50.7 ± 5.8^a^	53.1 ± 3.9^a^

^a^ *p* < 0.05 versus pre-op

^b^ *p* < 0.05 versus 6 months

^c^ *p* < 0.05 versus 12 months

The mean MOCART score was 42.8 ± 23.5 and 50.9 ± 24.9 points at 12 and 24 months, respectively, reflecting a significant improvement with time in terms of cartilage repair (Table 3, *p* < 0.05).

**Table 3:** MOCART score at 12 and 24 months follow-up

	12 months	24 months
Defect fill	8.0 ± 5.4	9.1 ± 5.8
Cartilage interface	3.3 ± 4.3	5.4 ± 4.0
Surface	2.6 ± 3	5.3 ± 3.4
Structure	3.0 ± 3.0	4.0 ± 3.0
Signal intensity	9.5 ± 10.6	11.3 ± 10.4
Subchondral lamina	2.8 ± 2.5	2.3 ± 2.5
Subchondral bone	3.9 ± 2.1	3.8 ± 2.2
Adhesions	5.0 ± 0.0	5.0 ± 0.0
Effusion	4.8 ± 1.1	4.9 ± 0.8
Total MOCART score	42.8 ± 23.5	50.9 ± 24.9^a^

^a^ *p* < 0.05 versus 12 months

**Fig. 1:**
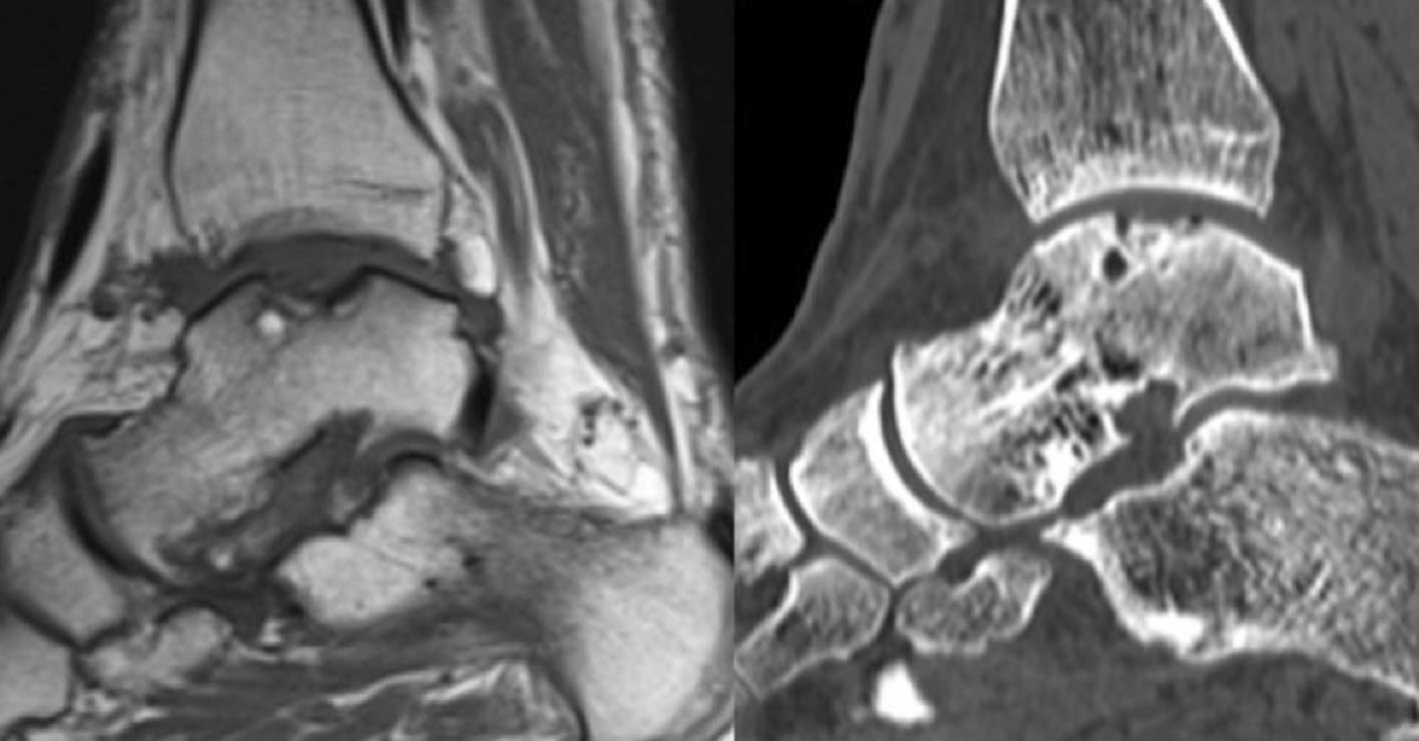
Osteochondral lesion on the talar dome: MRI compared with CT image. Sagittal view

**Fig. 2:**
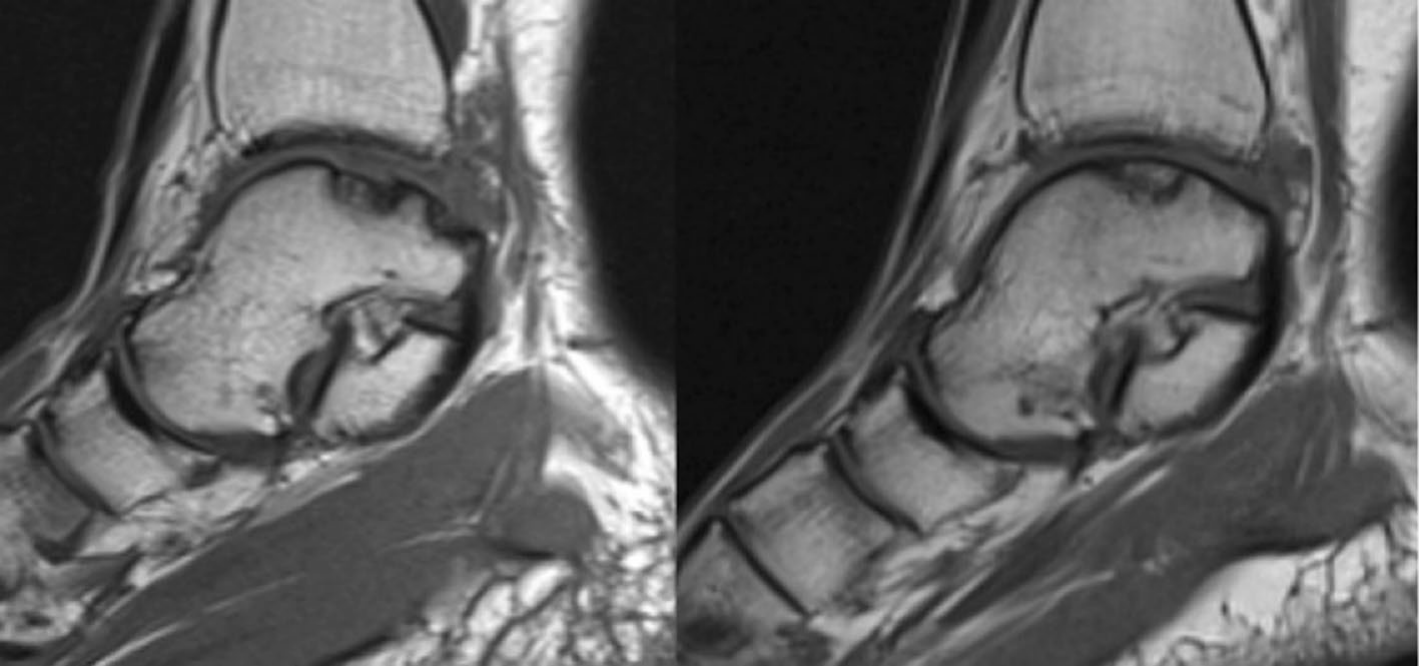
MRI comparing a OCLTs pre-operative and at 24 months follow-up. Sagittal view

Changes in lesion area were evaluated on both CT scan and MRI (Tables 4, 5). Data showed a progressive reduction of lesion area on CT scan until the last follow-up when the area was significantly smaller than the pre-operative value. On MRI, a significant reduction of the lesion was observed already after 12 months form surgery, with no further improvement at 24 months (Figs. 1, 2).

**Table 4:** Pre- and post-operative lesion area calculated on CT scan

	Pre-op	6 months	12 months	24 months
Mean ± SD (mm^2^)	111.1 ± 43.2	114.4 ± 37.9	94.5 ± 50.6	76.9 ± 38.1^a^
Max	194.0	182.7	217.2	174.0
Min	54.6	72.0	26.9	36.5

^a^ *p* < 0.05 versus pre-op

**Table 5:** Pre- and post-operative lesion area calculated on MRI

	Pre-op	6 months	12 months	24 months
Mean ± SD (mm^2^)	154.1 ± 93.6	120.0 ± 47.3	94.9 ± 62^a^	94.3 ± 61.3^a^
Max	352.0	234.1	258.3	244.5
Min	61.0	63.2	45.4	44.6

^a^ *p* < 0.05 versus pre-op

## Discussion

The most important finding of the present study is the effectiveness of the AT-AMIC^®^ technique for the treatment of OCLTs that yields improvement of both clinical and radiologic scores, together with an improvement in quality of life over a follow-up period of 24 months.

AMIC^®^ has been demonstrated to allow very satisfactory clinical and radiological results in knee and hip [7, 13, 21, 25] and, as recently published, comparable to other more complex and expensive cell-based cartilage repair techniques such as matrix autologous chondrocyte implantation (MACI) [8, 15].

OCLTs often lead to a degeneration of the subchondral bone, and the presence of cysts is not uncommon [23]. For this reason, and for other technical surgical issues, AMIC^®^ of the talus is most commonly performed and reported as an open surgical procedure.

In 2013, Walther and Martin reported the outcome of 42 patients with a minimum 2-month follow-up, showing a significant improvement of AOFAS score from 47.3 points to 88.3 points [25]. Subsequently, Valderrabano et al. [21] reported the clinical and radiographic results from 26 patients presenting OCLTs at a minimum of 24-months’ follow-up who underwent a modified AMIC^®^ technique using autologous bone grafting sealed by a collagen matrix without drilling of the subchondral bone. In 17 cases, ligament repair was associated with the cartilage repair procedure, while calcaneal osteotomy was performed in 16. The authors reported that the AMIC^®^ procedure was a safe treatment for OCLTs of the talus, with overall good radiological and clinical results reporting a mean post-operative MOCART score of 62 points where complete filling of the defect to the level of the surrounding cartilage was found in 35 % of patients, and complete filling with a hypertrophic cartilage layer in 50 % of patients. Moreover, 16 out of 19 patients returned to their sports activity practiced before the onset of their symptoms.

Recently, Kubosch et al. [11] reported results in seventeen patients with an osteochondral lesion of the medial talus who underwent surgery. Clinical and radiological assessment was performed after a mean follow-up of 39.5  ±  18.4 months, including AOFAS Score, Foot Function Index (FFI), VAS, MOCART Score, and T2 mapping. As well as finding a significant improvement in clinical parameters, a significant correlation was found between MOCART Score and the AOFAS Score and (*ρ*  =  0.57, *p* =  0.04) T2 relaxation time of the RT and the MOCART Score (*ρ*  =  0.59, *p* =  0.03). The researchers concluded that the one-step autologous subchondral cancellous bone grafting and AMIC leads to a significant reduction in post-operative pain and satisfying post-operative functional outcome in mid-term follow-up. MRI assessment demonstrated a good quality of regenerative tissue similar to the MRI ultrastructure of the surrounding cartilage.

Such minimally invasive surgical procedures represent undeniable advantages for the patient; in fact the open AMIC^®^ technique includes a malleolar osteotomy to achieve good talar dome exposure to successfully restore the dome anatomy for larger or more central/posterior and shoulder lesions. Possible complications of medial malleolus osteotomy are direct operative morbidity by injury to adjacent structures such as the posterior tibial tendon, the posterior tibial artery, the tibial nerve, or the healthy tibial cartilage; mid-term morbidity by malunion or nonunion of the osteotomy; and long-term morbidity by inducing or increasing the development of local cartilage degeneration and osteoarthritis and the necessity for hardware, which may also become symptomatic in an area with limited soft tissue envelope [5, 10].

The average complication rate in anterior ankle arthroscopy has been reported to be between 3.4 and 9 % [6, 24]. In our series, we had no complications related to the surgical technique, only one related to a hypertrophic reaction in a single patient that caused pain, impingement, and functional impairment, and required another arthroscopic surgery after 8 months after index procedure. In our cohort, no medial or lateral malleolar osteotomies were required, preserving the anatomy of the ankle. Moreover, the first arthroscopic step allows the status of the cartilage to be detected and accurately assessed.

In our study, CT and MRI were performed to evaluate patients at all time points. This work thus represents the first report, we are aware of, that assesses the outcome of AMIC^®^ using MRI in addition to CT. MRI has proven to overestimate the size of the lesions due to surrounding subchondral bone edema, confirming previously published work [14, 19]. Another important finding is that in the first six months, CT examinations showed an increase of lesion area due to the debridement and microfracturing performed during the surgical procedure but at last follow up of the lesion area was significantly reduced. In contrast, MRI showed a reduction of the injury already at 6 months after surgery with continuous improvement up to 2 years. To further evaluate cartilage repair, we assessed the MOCART score, which showed significant improvement at 24 months with respect to the 12-month follow-up. Our MOCART 24-month total mean score of 50.9 was lower than others who have reported in the existing literature [11, 21]; however, clinical- and patient-reported outcomes were comparable. Such differences have been discussed by several authors who have shown the difficulty in correlating MOCART with the clinical situation [1, 12, 16].

In 2013, Wiewiorksi et al. assessed cartilage quality in the same group of patients reported by Valderrabano [21] using delayed gadolinium-enhanced magnetic resonance imaging (dGEMRIC) and concluded that the biochemical properties of the cartilage repair tissue after AMIC^®^ repair of osteochondral lesions of the talus differ from normal hyaline cartilage [28].

The limitations of this study include the relatively small number of patients that did not allow for subpopulation analysis, and the lack of a control group, in particular with AMIC^®^ open procedure. Another limitation is represented by the lack of a dGEMRIC assessment of the repair tissue, which could have given useful information about tissue quality. However, the combination of MRI and CT information provided a good evaluation of the neo-cartilage and subchondral bone after the AT-AMIC^®^ surgical procedure. Another important limitation is the lack of sport activity assessment, particularly in young patients. Further studies will focus on the evaluation of the return to the sport in patients treated with AT-AMIC^®^ technique and autologous bone graft, also considering the type of sports activity (high or low impact) and time taken to return to sports.

## Conclusions

AT-AMIC^®^ has proven to be a safe and efficacious technique for the treatment of OCLTs, that yields excellent outcomes and that demonstrates clear improvement in pain and function, as well as radiological healing of the lesion. Orthopedic surgeons specialized in foot and ankle surgery should adopt the AT-AMIC^®^ technique for the treatment of osteochondral talar lesions, which proved to be effective and minimally invasive, avoiding malleolar osteotomy, with a low risk of complications.
